# Surgeons, sign your site!

**DOI:** 10.1186/s13037-017-0122-4

**Published:** 2017-04-04

**Authors:** Ted J. Clarke

**Affiliations:** Colorado Physician Insurance Companies (COPIC), 7351 E Lowry Blvd, Denver, CO 80230 USA

**Keywords:** Patient safety, sign your site, safety checklists, accountability

## Abstract

Please see related Short report: https://pssjournal.biomedcentral.com/articles/10.1186/s13037-017-0125-1

## Culture eats protocol for lunch^1^

In 2010, COPIC data was used to publish a study on wrong site/wrong patient surgery in the era of the Universal Protocol [1]. Although this “never event” is thankfully rare, it proved quite alarming when the publication on 27,000 reported adverse incidents revealed 107 wrong-site and 25 wrong-patient procedures being performed in the current era of standardized surgical safety checklists [1]. The publication was intended as a “call to action” for surgeons to embrace the patient safety movement as a non-negotiable surgical responsibility [2]. By putting the patient at the center of all activities performed by the health care team and by having surgeons, nurses, and other health care givers treat the patient with the same due diligence and respect that each would expect from their caregivers, patient safety and quality improvement in health care can move to a new level.

The initial uproar created by this paper [3, 4] was soon lost amidst the quiet library shelves and computer files of various journals and hard drives. Unfortunately, our COPIC trend in wrong-site/wrong-patient surgeries continued after the paper’s publication along the unacceptable trend line of 10–15 such incidents per year (Michael Victoroff, MD, personal communication, January 2017). Because of this trend, COPIC and the Colorado Hospital Association embarked on a study to drill down on the persistence of this seemingly avoidable event. Walter Biffl led a team of investigators that catalogued 854 operating room observations at ten different Colorado hospitals [5]. Each hospital required compliance of a modified surgical WHO checklist, and the findings of this study showed significant variability in the utilization of this safety tool. The dominant factor in compliance was the surgeon and the surgical team’s “buy-in” as to the importance of the checklist in reducing errors and improving outcomes [5].

## The risk of delegating site marking to non-surgeons

In the current issue of *Patient Safety in Surgery*, Schäfli-Thurnherr and colleagues report an observational study at a Swiss teaching hospital testing the provocative hypothesis that that nurses are capable of safely marking the preoperative surgical site without compromising safety [6]. The rationale given for this study was the “non-feasible” aspect of requiring surgeons to sign their patients’ surgical site in the setting of same-day surgery. Following a brief training period for select nurses at the involved institution, the authors claim that no wrong-site surgeries occurred during a nearly 3-year observational period [6]. The report concludes that nurses and surgeons felt positive about the program, and that signing the surgical site can be safely delegated to non-surgeons [6].

As an advocate for patient safety, I found the paper by Schäfli-Thurnherr et al. [6] to be very disturbing. I attempted to imagine the pre-op holding area where it was “not feasible” for surgeons to sign the correct surgical site for an upcoming operation on their patient. Visions of the chaotic war-zone medical care in a “MASH unit” came to my mind, which however seemed somewhat incongruous in a renowned Swiss institution that provides high-quality surgical care. Although perhaps facilitating patient flow, the Swiss team seemingly misses the point of having the surgeon sign the correct surgical site. Signing the site allows surgeons to examine and confirm the proposed surgical field. This process also allows the patient to see and feel the site of the future scar that he will wear for the rest of his life. The patient and family frequently have several unanswered questions prior to surgery that only the surgeon can coherently address. The pre-operative discussion between surgeon and patient, accompanied by the ritual of signing the site, helps alleviate the patient of the fears and anxieties that accompany every surgery, as part of the preoperative shared decision-making process [7].

Characterizing the WHO checklist as “not feasible” suggests that the surgeon’s signing of the site is an impediment to efficient patient flow. So let me ask the provocative question: Are surgical safety checklists merely a check to be marked for the compliance imposed by a third party regulator? To delegate this crucial duty to non-surgeons suggests the true value of this patient-centered process is not embraced or not understood in its significance as part of an encompassing patient safety culture. Would the patient be equally satisfied with this change in protocol? The culture of patient safety, a culture that puts the patient at the center of all activities by the health care team, has seemingly failed to evolve in the context of this most recent study [6].

## Conclusions

Until surgeons embrace the patient safety movement, we will continue to witness preventable wrong-side and wrong-patient events. Throughout the surgical process, the surgeon should put himself in the patient’s position. The surgeon must continuously advocate for the patient as he would advocate for himself [2]. The WHO checklist has been shown to dramatically improve surgical outcomes and lack of adherence to the checklist will continue placing our patients at risk of preventable complications and adverse outcomes [8]. Yes, a surgeon can delegate the signing of the correct surgical site to another health care provider, but in doing this, the surgeon will relinquish personal accountability and send the wrong message to his team. The surgeon is the team leader and should act as an unquestioned and credible role model for the well-being of his patient. Only when the surgeon is putting the needs of his patient first will we see a true transformation to a culture of patient safety. For the sake of your patients, surgeons – sign your site!

## Footnotes

1 Quote adapted from Peter F. Drucker (1909–2005)
